# Supplementary material for: The Diet Quality and Nutrition Inadequacy of Pre-Frail Older Adults in New Zealand
Source: Nutrients. 2021 Jul 13;13(7):2384. doi: 10.3390/nu13072384 (PMC8308886; doi:10.3390/nu13072384)
Supplement: Supplementary file 1 [file nutrients-13-02384-s001.zip › nutrients-1280775-supplementary.pdf]

Supplementary Materials

Table S1. Diet Quality Index-International (DQI-I) scoring system and scores for pre-frail older adults.

Component	Maximum Score	Scoring criteria per day			All	Male	Female	*p*-value
n					465	191	274	
**Variety**	**20**				13.50 (11.00 – 15.50)	13.50 (11.00 – 16.50)	13.00 (11.00 – 15.00)	*0.044*
Overall food group variety *(meat/poultry/fish/eggs/nuts and seeds[1]; dairy/beans; grain; fruit; vegetable)*	15	≥ 1 serv from each unique FG of the five listed = 3			10.50 (9.00 – 12.00)	10.50 (9.00 – 12.00)	10.50 (9.00 – 12.00)	0.187
Within group variety *(meat, poultry, fish, dairy, beans, eggs, nuts/seeds[1])*	5	1 source (≥ 0.5 serv) = 1<br>2 unique sources = 3<br>≥3 unique sources = 5			3.00 (2.00 – 4.00)	3.00 (2.00 – 4.00)	3.00 (2.00 – 4.00)	0.136
**Adequacy**	**40**	**TEI level**			29.38 (26.60 – 32.28)	28.97 (26.33 – 32.58)	29.75 (26.72 – 32.14)	0.142
		**1700 kcal**	**2200 kcal**	**2700 kcal**				
Vegetable[2]	5	% attainment of below thresholds per energy level x 5			2.75 (1.75 – 3.85)	2.73 (1.51 – 3.68)	2.80 (1.97 – 4.01)	0.735
		3 serv	4 serv	5 serv				
Fruit[2]	5	% attainment of below thresholds per energy level x 5			2.96 (1.61 – 4.28)	2.54 (1.3 – 4.08)	3.26 (1.90 – 4.46)	0.016
		2 serv	3 serv	4 serv				
Grain	5	% attainment of below thresholds per energy level x 5			2.87 (2.05 – 3.85)	3.05 (2.18 – 3.94)	2.68 (1.87 – 3.70)	0.035
		6 serv	9 serv	11 serv				
Fibre (g)	5	% attainment of below thresholds per energy level x 5			4.22 (3.46 – 4.83)	4.36 (3.39 – 4.96)	4.15 (3.48 – 4.75)	0.083
		20 g	25 g	30 g				
Protein (g)	5	% attainment of contribution to energy intake (≥ 10% TEI) x 5			5.00 (5.00 – 5.00)	5.00 (5.00 – 5.00)	5.00 (5.00 – 5.00)	-
Iron (mg)	5	% attainment of RDI[5] (8 mg) x5			4.91 (4.26 – 5.00)	5.00 (4.69 – 5.00)	4.61 (4.03 – 5.00)	*<0.001*
Calcium (mg)	5	% attainment of RDI (1300 mg) x5			2.71 (2.01 – 3.54)	2.87 (2.15 – 3.63)	2.56 (1.96 – 3.46)	0.007
Vitamin C (mg)	5	% attainment of RDI (45 mg) x5			4.89 (3.53 – 5.00)	4.87 (3.16 – 5.00)	4.90 (3.63 – 5.00)	0.969
**Moderation**	**30**	**6**	**3**	**0**	12.00 (9.00 – 15.00)	12.00 (9.00 – 15.00)	12.00 (10.50 – 15.00)	*0.002*
Total fat (%TEI)	6	≤20%	>20-30%	>30%	1.50 (0.00 – 1.50)	1.50 (0.00 – 1.50)	0.75 (0.00 – 1.50)	0.729
Saturated fat (%TEI)	6	≤7%	>7-10%	>10%	0.00 (0.00 – 1.50)	0.00 (0.00 – 1.50)	0.00 (0.00 – 1.50)	0.649
Cholesterol (mg)	6	≤300	>300-400	>400	6.00 (4.50 – 6.00)	6.00 (3.00 – 6.00)	6.00 (4.50 – 6.00)	-
Sodium (mg)	6	≤2400	>2400-3400	>3400	6.00 (4.50 – 6.00)	4.50 (3.00 – 6.00)	6.00 (4.50 – 6.00)	-
Empty calorie foods[3] (%TEI)	6	≤3%	>2-10%	>10%	0.00 (0 .00 – 0.00)	0.00 (0 .00 – 0.00)	0.00 (0 .00 – 0.00)	0.750

Component	Maximum Score	Scoring criteria per day		All	Male	Female	*p*-value
**Overall balance**	**10**			4.67 (3.50 – 5.67)	4.67 (3.67 – 6.00) [a]	4.67 (3.46 – 5.50) [b]	0.261†
Macronutrient ratio	6	Deviation %TEI	Score	4.00 (3.00 – 5.00)	4.00 (3.00 – 5.00)	4.00 (2.67 – 4.75)	0.832
(carbohydrate: protein: fat[4])		0% [45-65:15-25:20-35]	6				
		5% [43-68:14-26:19-37]	4				
		10% [41-72:13-27:18-39]	2				
		>10% [otherwise]	0				
Fatty acid ratio	4	PUFA/SFA & MUFA/SFA	Score	1.00 (0.00 – 1.00)	1.00 (0.00 – 1.00)	1.00 (0.83 – 1.00)	0.745
(PUFA: MUFA: SFA)		1-1.5 both	4				
		0.8-1.7 both	2				
		Otherwise	0				
**Total**	**100**			60.27 (53.96 – 64.69)	59.27 (53.15 – 67.87) [c]	60.83 (54.33 – 64.64)	*0.042*

Values are expressed as median (IQR). Significance was set at $p < 0.05$ for variety, adequacy, moderation, overall balance, and total DQI-I score. Bonferroni adjusted significance was used at $p < 0.00294$ for the 17 comparisons of subcomponents. *P*-values of significance between sexes are italicised. † *P*-value determined by independent samples T-test while all other *p*-values were derived from non-parametric samples tests. Normally distributed values mean (SD) as follows: [a] overall balance score 4.68 (1.47), [b] overall balance score 4.52 (1.53), [c] DQI-I total score 58.84 (8.62).

Abbreviations: food group (FG), serving (serv), total energy intake (TEI), polyunsaturated fatty acids (PUFA), monounsaturated fatty acids (MUFA), saturated fatty acids (SFA), recommended dietary intake (RDI) [31].

Adaptations to the DQI-I: [1] nuts and seeds added as a component of protein-based food group as seen in New Zealand guidelines [31]; [2] starchy vegetables, fruit juice and dried fruit servings count capped at one serve as seen in New Zealand guidelines [31]; [3] non-essential, energy-dense, nutritionally-deficient foods in New Zealand (NEEDNT) food list utilised and adapted for older people e.g. removed whole milk and yoghurt from list [32]; [4] adapted DQI-I ratio to meet the New Zealand acceptable macronutrient distribution range (AMDR) [31].

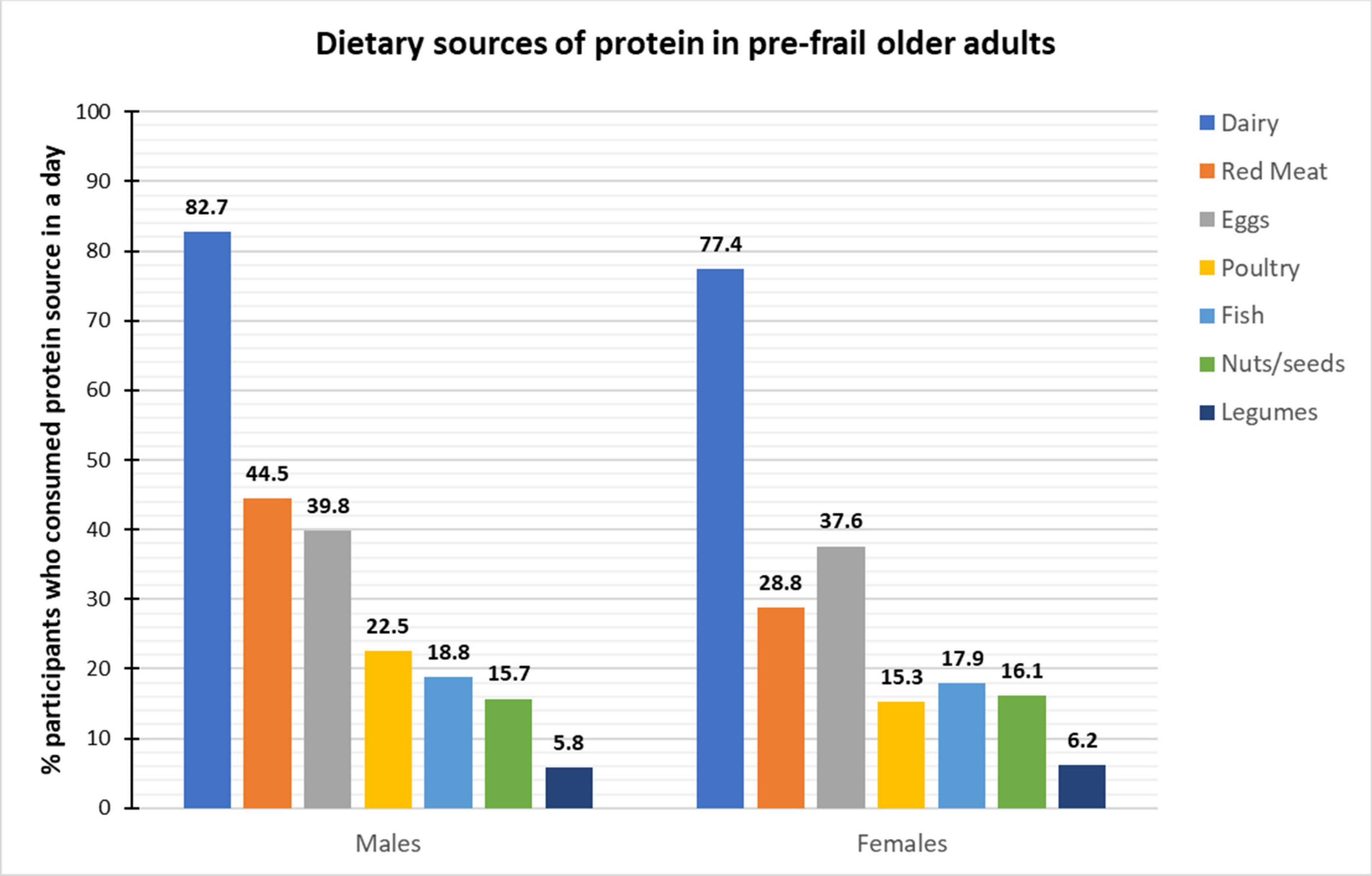

Figure S1. Protein sources according to the Diet Quality Index-International (DQI-I) in pre-frail older adults sorted in descending order of percentage consumed for males.

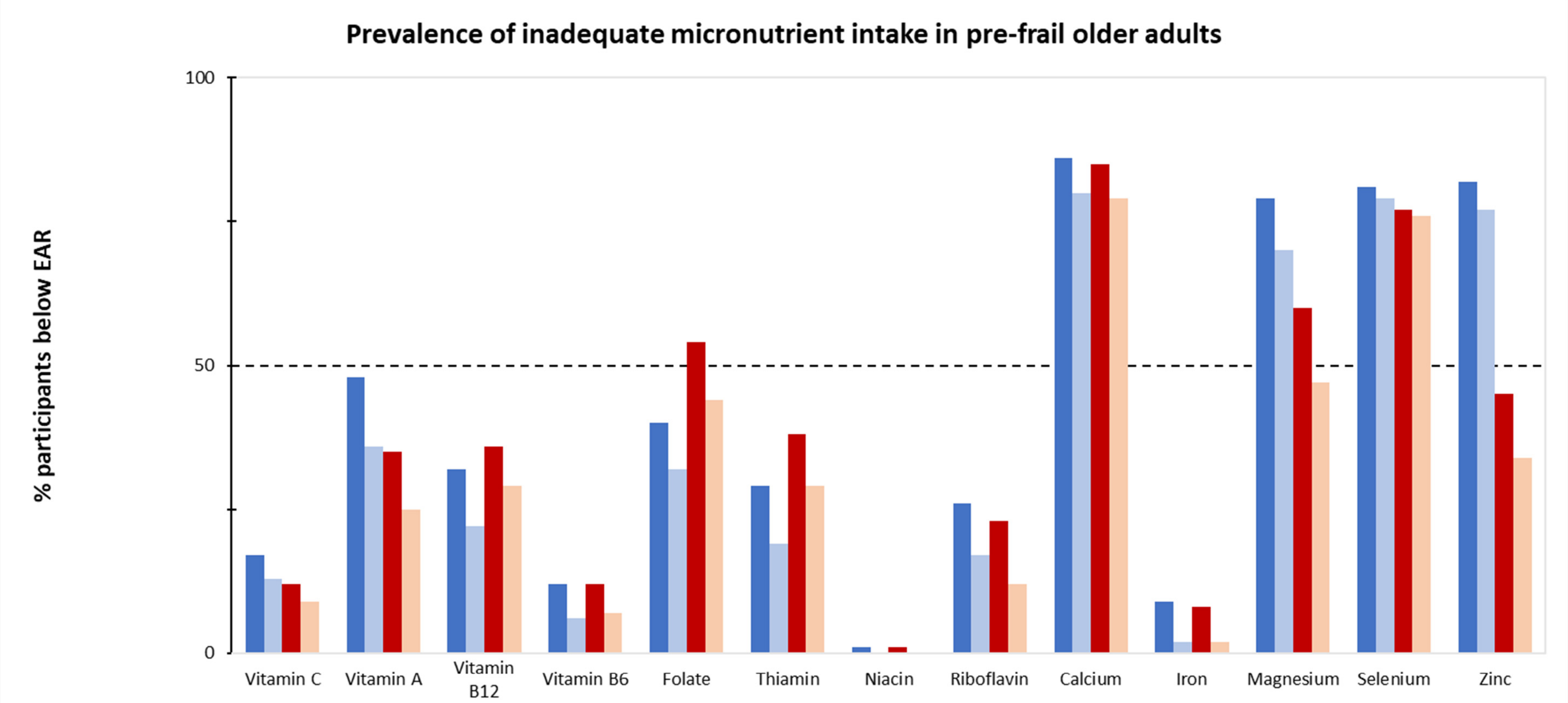

	Vitamin C	Vitamin A	Vitamin B12	Vitamin B6	Folate	Thiamin	Niacin	Riboflavin	Calcium	Iron	Magnesium	Selenium	Zinc
Male All	17	48	32	12	40	29	1	26	86	9	79	81	82
Male Excluding LER	13	36	22	6	32	19	0	17	80	2	70	79	77
Female All	12	35	36	12	54	38	1	23	85	8	60	77	45
Female Excluding LER	9	25	29	7	44	29	0	12	79	2	47	76	34

Figure S2. Bar graph demonstrating prevalence of inadequate micronutrient intake among pre-frail older adults according to estimated average requirement (EAR) recommendations.

Note: Due to EAR unavailability for Vitamin D and E, these micronutrients alongside sodium are excluded from the graph. Data is presented according to sex and comparisons made between all participants versus low energy reporters (LER) excluded.

Table S2. Summary of nutritional elements by sex in comparison to guidelines and the 2008/9 nutrition survey findings for older adults.

Nutrient	Unit per day	Male (*n* = 191)	Female (*n* = 274)	*p*-value	Guidelines for 70+				2008/9 survey in 75+ (or 71+)	
					Male		Female		Male	Female
					EAR	RDI	EAR	RDI		
Energy intake	kJ	7147.46 (5926.08 – 8586.62) [a]	5777.49 (4802.24 – 7232.44)	*<0.001*	-		-		7477	5569
	kcal	1702.34 (1410.41 – 2041.21) [b]	1373.24 (1143.99 – 1723.30)	*<0.001*	-		-		-	-
	kcal/kg BW	21.07 (16.07 – 25.75)	19.73 (15.19 – 25.09)	0.157	25 – 30		25 – 30		-	-
Carbohydrate	g	195.98 (158.67 – 243.25)	164.83 (129.91 – 200.70)	*<0.001*	-		-		213	169
	% TEI	46.80 (42.01 – 53.45) [c]	47.69 (42.55 – 52.60)	0.142	45 – 65		45 – 65		48	48
Total sugars	g	85.18 (60.64 – 119.06)	78.03 (55.20 – 106.07)	0.122	-		-		(100)	(82)
Dietary Fibre[1,2]	g	20.97 (15.78 – 26.15)	18.20 (14.74 – 22.19)	0.004	30		25		19.5	16.6
Protein	g	67.47 (55.27 – 82.09) [d]	56.37 (44.51 – 67.05)	*<0.001*	65	81	46	57	72.7	57.7
	g/kg BW	0.81 (0.64 – 1.04)	0.78 (0.60 – 0.99)	0.509	1.07		0.94		-	-
	% TEI	15.67 (13.50 – 18.13)	15.94 (13.51 – 18.26)	0.735	15 – 25		15 – 25		16	16
Fat	g	63.79 (48.46 – 81.41)	54.11 (38.86 – 69.87)	0.002					63	51
	% TEI	33.61 (27.63 – 38.69) [e]	34.32 (28.75 – 39.45)	0.366	20 – 35		20 – 35		31	33
Saturated Fat	g	25.28 (17.96 – 33.22)	20.38 (14.44 – 29.03)	*<0.001*	-		-		24	20
	% TEI	13.30 (10.94 – 15.95) [f]	13.18 (10.66 – 16.08)	0.969	(with trans) < 10		(with trans) < 10		12	12
Omega-6[1]	g	4.39 (2.95 – 6.60)	3.80 (2.42 – 5.41)	0.013	13		8		-	-
Omega-3[1]	g	0.67 (0.43 – 1.01)	0.58 (0.39 – 0.82)	0.083	1.3		0.8		-	-
Omega 6:3 ratio		7.18 (5.08 – 10.8)	6.59 (5.02 – 9.17)	0.258	-		-		-	-
Cholesterol	mg	212.57 (120.12 – 305.19)	163.95 (111.50 – 251.28)	*0.001*	-		-		(244)	(193)
Alcohol	g	0.00 (0.00 – 12.48)	0.00 (0.00 – 3.86)	0.006	-		-		-	-
Vitamin D[1]	μg	2.92 (1.81 – 4.61)	2.41 (1.48 – 4.43)	0.004	15		15		-	-
Vitamin C	mg	71.58 (38.08 – 106.37)	77.75 (44.23 – 115.08)	0.474	30	45	30	45	(96)	(89)
Vitamin E (α-TE)[1]	mg	7.23 (5.07 – 9.31)	6.39 (4.89 – 8.16)	0.035	10		7		(10.2)	(8.5)
Vitamin A, (RE)	μg	637.76 (421.43 – 856.46)	612.67 (408.30 – 845.30)	0.678	625	900	500	700	(851)	(768)
Vitamin B12	μg	2.77 (1.83 – 4.01)	2.39 (1.64 – 3.47)	0.035	2.0	2.4	2.0	2.4	5.0	2.3
Vitamin B1 (Thiamin)	mg	1.42 (0.95 – 2.34)	1.01 (0.76 – 1.44)	*<0.001*	1.0	1.2	0.9	1.1	(1.4)	(1.1)

Nutrient	Unit per day	Male (n = 191)	Female (n = 274)	p-value	Guidelines for 70+				2008/9 survey in 75+ (or 71+)	
					Male		Female		Male	Female
					EAR	RDI	EAR	RDI		
Vitamin B2 (Riboflavin)	mg	1.79 (1.27 – 2.15)	1.53 (1.12 – 2.05)	*0.001*	1.3	1.6	1.1	1.3	(1.8)	(1.5)
Vitamin B3 (NE)	mg	35.70 (26.72 – 43.92) [g]	30.29 (23.49 – 37.36)	*0.001*	12	16	11	14	(31.4)	(23.3)
Vitamin B6	mg	2.4 (1.76 – 3.10)	2.08 (1.57 – 2.93)	0.004	1.4	1.7	1.3	1.5	(1.6)	(1.3)
Folate (DFE)	µg	354.57 (242.71 – 560.75)	292.47 (192.23 – 427.33)	0.007	320	400	320	400	-	-
Selenium	µg	36.4 (27.2 – 52.48)	32.76 (23.24 – 48.58)	0.054	60	70	50	60	(52.0)	(39.5)
Calcium	mg	747.15 (559.94 – 950.53)	664.39 (510.10 – 907.71)	0.007	1100	1300	1100	1300	698	658
Iron	mg	10.9 (8.38 – 14.01)	8.33 (6.71 – 10.60)	*<0.001*	6	8.0	5	8.0	(11.4)	(8.9)
Magnesium	mg	284.38 (239.27 – 339.82)	244.07 (202.51 – 300.82)	*<0.001*	350	420	265	320	-	-
Zinc	mg	8.56 (6.60 – 10.49)	6.85 (5.35 – 8.79)	*<0.001*	12.0	14	6.5	8	9.3	7.4
Sodium[1]	mg	2140.05 (1701.16 – 2800.03)	1595.14 (1251.57 – 2160.42)	*<0.001*	460 – 920		460 – 920		-	-
Water[1]	ml	2139.60 (1727.99 – 2584.90) [h]	2089.45 (1703.58 – 2458.80)	0.546	3.4L (or 2.6L fluids only)		2.8L (or 2.1L fluids only)		-	-
	ml/kg BW	25.80 (19.48 – 32.89)	28.30 (22.88 – 35.44)	0.011	35		35		-	-

Nutrient reference values and guidelines derived from the 2013 Food and nutrition guidelines for healthy older people [31] and the Dietitians New Zealand Clinical Handbook 2016 [33]. 2008/9 New Zealand adult nutrition survey findings are described in detail in the Ministry of Health report [28]. Bonferroni adjusted significance set at α = 0.00179 for 28 comparisons; Italicized p-values are significant at this level. P-values were derived from non-parametric samples tests. Normally distributed values, mean (SD) as follows: [a] energy 7321.49 (2128.95) kJ, [b] energy 1743 (508.3) kcal, [c] carbohydrate 47.31 (7.93) %TEI, [d] protein 68.47 (20.41) g, [e] fat 33.26 (7.26) % TEI, [f] saturated fat 13.36 (3.72) % TEI, [g] vitamin B3 35.83 (11.37) mg, [h] water 2188.51 (621.58) ml.

Abbreviations: estimated average requirement (EAR), recommended dietary intake (RDI), body weight (BW), α-tocopherol equivalents (α -TE); retinol equivalents (RE), niacin equivalents (NE), dietary folate equivalents (DFE). [1] Adequate intake. [2] Dietary fibre was measured using AOAC from the New Zealand FOODfiles 2016 database and the Englyst method from the Nutrient Databank from Public Health England.

Table S3. Multiple forward linear regression models showing the association of demographic and health variables with low energy reporters compared to plausible reporters.

Model	R^2 adjusted (n = 464)	Variables	B	95% Confidence intervals for B	p-value
1	0.139, $p < 0.001$	Constant	-0.697	-0.931, -0.462	<0.001
		BMI	0.036	0.028, 0.044	<0.001
2	0.153, $p < 0.001$	Constant	-0.228	-0.618, 0.163	0.253
		BMI	0.034	0.026, 0.042	<0.001
		Total DQI-I (/100)	-0.007	-0.012, -0.002	0.004

Regression models conducted using multiple forward linear regression. All models were adjusted for age, sex, marital status, education, living arrangement, deprivation, medical conditions, vision, hearing, medications, supplements, alcohol, NEADL, and total DQI-I score except for total DQI-I score in model 2.
